# Editorial: Bioinformatics and Biostatistics Applications in Tobacco Smoking Research

**DOI:** 10.3389/fpubh.2018.00366

**Published:** 2018-12-11

**Authors:** Mohammed A. Ibrahim Al-Obaide, Abdel-Salam G. Abdel-Salam, Nisreen DaifAllah AL-Hmoud, Hayfa H. Hassani, J. P. Verma

**Affiliations:** ^1^Texas Tech University Health Sciences Center, Amarillo, TX, United States; ^2^Department of Mathematics, Statistics and Physics, Qatar University, Doha, Qatar; ^3^Princess Sumaya University for Technology, Amman, Jordan; ^4^Department of Biology, College of Science, University of Baghdad, Baghdad, Iraq; ^5^Department of Sport Psychology, Lakshmibai National Institute of Physical Education, Gwalior, India

**Keywords:** bioinformatics, biostatistics, tobacco smoking behavior, biostatistics applications, smoking research

The Bioinformatics, Biostatistics, and Genomics are examples of the multidisciplinary and interdisciplinary blend of scientific fields merging two or more specialties help express robust conclusion of respective research activity. The selected Research Topic “Bioinformatics and Biostatistics Applications in Tobacco Smoking Research” is of great interest because of smoking being an important public health concern. The articles in this Research Topic provided recent advancement in the understanding of the impact of tobacco smoking on health. The articles published in two Frontiers Journals that presented examples of the range of subjects covered associated with health effects of tobacco smoking. Two of the five accepted articles are on “Perspective” and “Mini-review” while remaining three are on “Original” research findings. Ibrahim et al. showed the prevalence of tobacco smoking and the negative impact on health of the people of Iraq. The authors demonstrated tobacco use was the main risk factor associated with cancer and chronic obstructive pulmonary diseases, which are the leading cause of morbidity and mortality in Iraq. Furthermore, the study further revealed a link between smoking and psychological problems and postwar conflicts continued since 1980. The second study, a mini-review, showed the impact of the smoke from tobacco smoking comprises more than 4,000 compounds, and dozens with carcinogenic activities potentially cause mutational changes in the DNA sequences and DNA methylation profiles (Al-Obaide et al.). The mutational changes caused by tobacco smoking can produce single nucleotide polymorphisms associated with various types of cancers. For example, gene polymorphisms of the CYP1A1 gene (CYP1A1m1, T6235C, and CYP1A1m2, A4889G) found significantly associated with an elevated risk of breast cancer in women from Iraq (Naif et al.). According to a recent study, metformin is associated with nicotine cessation by activation of the AMPK signaling pathway (1). The AMPK pathway has a vital function in the control of cell growth and metabolism (2). The fourth study proposed the association of prolific proteomic changes due to the therapeutic mechanisms of metformin on breast cancer cells (Al-Zaidan et al.). The fifth study presented an unexplored regulatory mechanism involved in kynurenine 3-monooxygenase gene, KMO, expression. The KMO locus associated with nicotine initiation and addiction. The study showed the function of the uncharacterized ncRNA, LOC105373233 locus in the regulation of KMO expression (Aziz et al.). KMO has multifaceted functions, and changes in KMO expression or activity may contribute to the development of neurodegenerative, neuropsychiatric, and neurodevelopmental diseases (3). The ncRNAs characterized by selective targeting of genes and consequently can have a detrimental impact on health (4). Exposure to smoke from cigarette can cause aberrant expression and function of ncRNA (5, 6). Our current understanding of the regulatory mechanisms of ncRNA includes the activation or inhibition of transcription and alteration of chromatin state, which points to the harmful consequences of tobacco smoke chemicals on the ncRNA regulatory function of gene expression. In summary, all five articles are of diverse yet with motivating views on the impact of tobacco smoking on health. The authors investigated and discussed the molecular mechanisms that link mutations and specific genes to tobacco use and diseases. The topic articles provided insight of the health effects prevalent among cigarettes smokers, and required regulatory measures for its prevention since tobacco smoking is a widespread behavior in both developed and developing countries.

## Author Contributions

All authors listed have made a substantial, direct and intellectual contribution to the work, and approved it for publication.

### Conflict of Interest Statement

The authors declare that the research was conducted in the absence of any commercial or financial relationships that could be construed as a potential conflict of interest.
